# Supplementary material for: Mott gap collapse in lightly hole-doped Sr2−xKxIrO4
Source: Nat Commun. 2020 May 22;11:2597. doi: 10.1038/s41467-020-16425-z (PMC7244596; doi:10.1038/s41467-020-16425-z)
Supplement: Supplementary file 1 — Supplementary Information [file 41467_2020_16425_MOESM1_ESM.pdf]

Supplementary Information: Mott gap collapse in lightly hole-doped $Sr_{2-x}K_xIrO_4$

J. N. Nelson,[1] C. T. Parzyck,[1] B. D. Faeth,[1] J.K. Kawasaki,[1,2,3,4] D. G. Schlom,[2,3] and K. M. Shen[1,3,*]

[1]*Laboratory of Atomic and Solid State Physics, Department of Physics, Cornell University, Ithaca, New York 14853, USA*

[2]*Department of Materials Science and Engineering, Cornell University, Ithaca, New York 14853, USA*

[3]*Kavli Institute at Cornell for Nanoscale Science, Ithaca, New York 14853, USA*

[4]*Current Address: Department of Materials Science and Engineering, University of Wisconsin, Madison, Wisconsin 53706, USA*

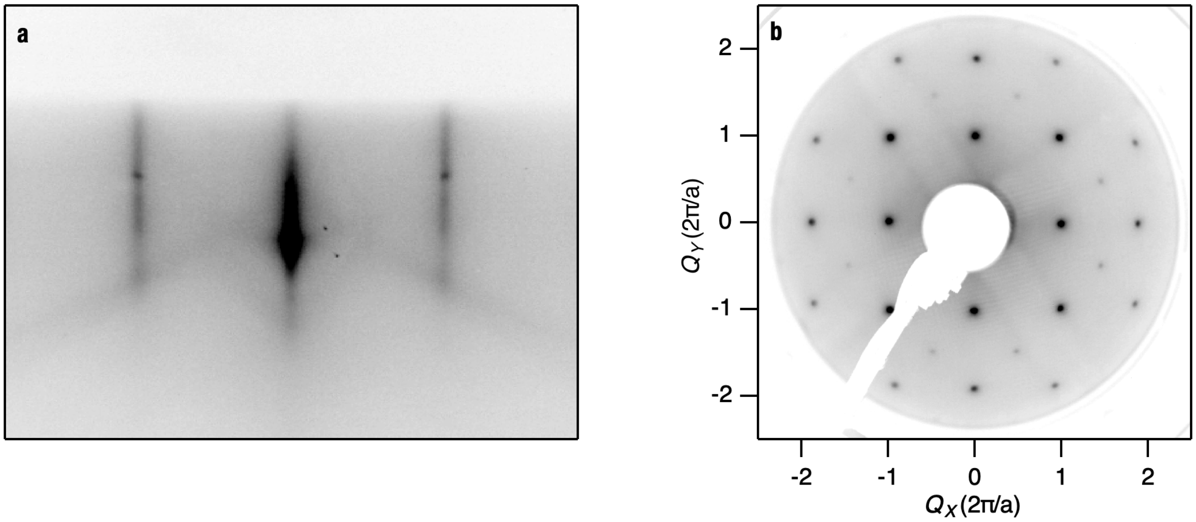

Supplementary Figure 1. Electron diffraction measurements of $Sr_{1.93}K_{0.07}IrO_4$. **a** RHEED image along the [110] azimuth showing sharp streaks indicating smooth film growth. **b** 200 eV LEED image showing a well ordered surface with half order spots indicating a $\sqrt{2} \times \sqrt{2}R45^{\circ}$ reconstruction.

Supplementary Note 1: Film Growth and Characterization

Thin films of Sr_2IrO_4 were grown on a grounding layer of $SrIrO_3$ on (001)-oriented single-crystal $(LaAlO_3)_{0.3}$ $(SrAl_{1/2}Ta_{1/2}O_3)_{0.7}$ (LSAT) substrates by reactive oxide molecular-beam epitaxy (MBE) in a dual-chamber Veeco GEN10 system. A background partial pressure of 1×10^{-6} torr of distilled ozone (80% O_3 + 20% O_2) was used and the substrate temperature (as measured by an optical pyrometer with a measurement wavelength of 980 nm) was 850 °C for the Sr_2IrO_4 growth and 640 °C for the $SrIrO_3$ growth. A Sr flux of 1×10^{13} atoms $cm^{-2}s^{-1}$ was evaporated from a low temperature effusion cell, and Ir was supplied by a electron beam evaporator. Ir and Sr were codeposited during the growth with the non stoichiometric flux ratio of Sr/Ir = 1.15 for the Sr_2IrO_4 and 0.82 for the $SrIrO_3$ placing the growths in an absorption controlled regime shown to be successful for the growth of other Ruddlesden-Popper systems[1–8]. In situ reflection high-energy electron diffraction (RHEED) was used to monitor the film growth. A representative image shows sharp streaks indicating a smooth surface of $Sr_{1.93}K_{0.07}IrO_4$ (supplementary fig. 1). Fluxes were calibrated using a quartz crystal microbalance (QCM) before growth and by RHEED oscillations during growth of the $SrIrO_3$. Growth of the Sr_2IrO_4 did not display RHEED oscillations, but produced a similar surface quality likely because Sr_2IrO_4 grows in step-flow mode for the growth

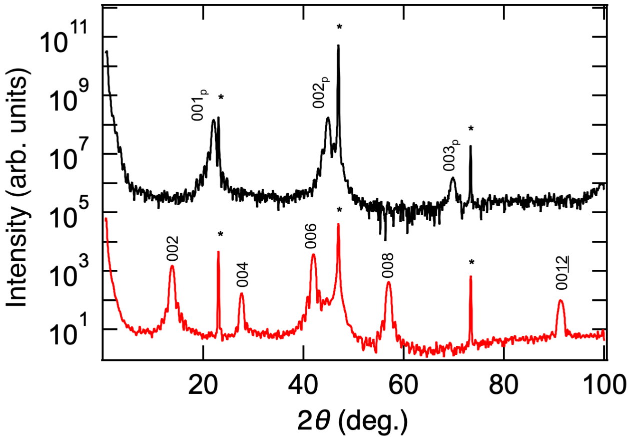

Supplementary Figure 2. X-ray diffraction 2θ scans exhibiting clear thickness fringes from both Sr_2IrO_4 (red) and $SrIrO_3$ (black), peaks due to the $(LaAlO_3)_{0.3}$ $(SrAl_{1/2}Ta_{1/2}O_3)_{0.7}$ (LSAT) substrate are indicated by *.

conditions used.

The structure of the resulting films was characterized through x-ray diffraction with Cu Kα. Supplementary figure 2 shows 2θ scans on representative Sr_2IrO_4 and $SrIrO_3$ samples indicating that both films are phase pure and exhibit thickness fringes, indicating high quality crystalline growth with sharp interfaces.

In total, eleven $Sr_{2-x}K_xIrO_4$ thin films were synthesized for this study. For these eleven samples, the post-growth K substitutional diffusion conditions were varied significantly, including (1) the amount of K deposited on the surface was varied by a factor of 40; (2) the concentration of ozone used in the annealing step (both 10% and 80%); (3) and the amount of time during the vacuum annealing (24 to 40 min) and ozone annealing (20 to 55 min). Despite varying all these conditions, we have found that of the seven samples that yielded high quality ARPES spectra which can be reliably analysed, all seven samples give the same extracted hole doping concentration of 7$\pm$ 2% (from Luttinger volume) and a chemical potential shift of $\Delta\mu$ =-0.4 $\pm$ 0.1 eV. It is possible that a 7% doping may be energetically favoured in the substitutional diffusion process, or that this represents the solubility limit of K in Sr_2IrO_4 .

The chemical content of the film was measured using x-ray photoemission spectroscopy with a Mg k-alpha (hν = 1254 eV) Scienta-Omicron DAR 400 twin-anode X-ray source before and after substitutional diffusion of K on the same sample, shown in Supplementary Fig. 3. This

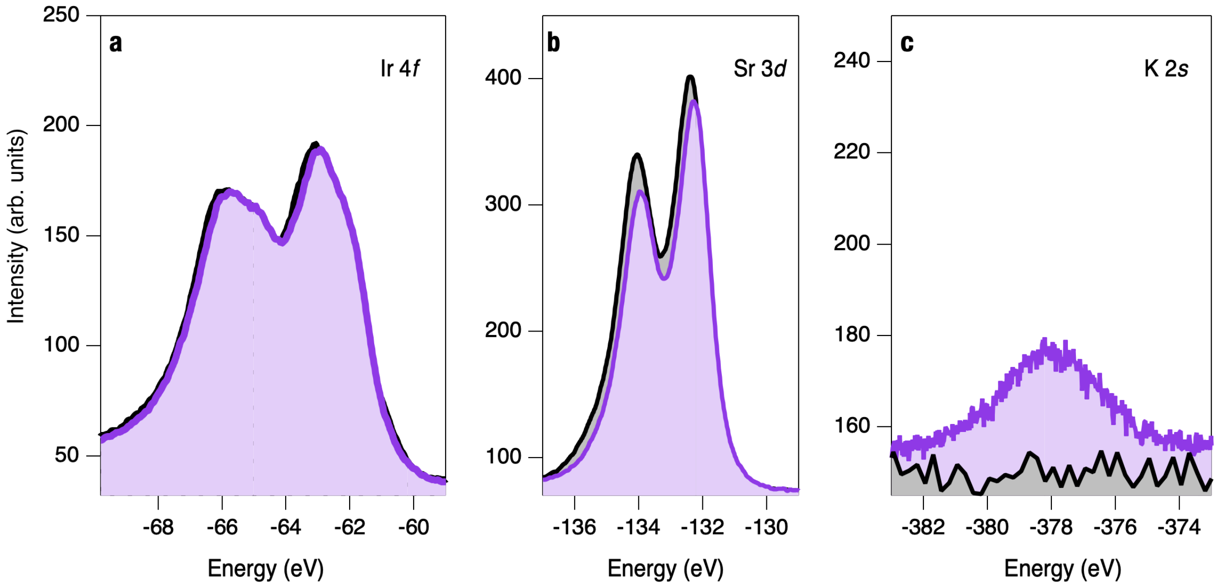

Supplementary Figure 3. Normalized x-ray photoemission spectra of undoped Sr_2IrO_4 (black) and $Sr_{1.93}K_{0.07}IrO_4$ (purple). **a** Ir 4*f* peak, **b** Sr 3*d* peak and **c** K 2*s* peak.

demonstrates that the substitutional diffusion process results in increased K content and decreased Sr content in the region of the sample probed by photoemission. We do not see any signature of the $SrIrO_3$ in photoemission measurements because the thickness of Sr_2IrO_4 is much larger than the inelastic mean free path of electrons with a kinetic energy of 21 eV, 41 eV and 1254 eV[9].

Supplementary Note 2: Control Experiment

In order to confirm that the observed change in electronic structure is due to K dopants and not an unintentional change of oxygen stoichiometry we perform a control experiment where a Sr_2IrO_4 sample is annealed at a temperature of 300 °C in vacuum, followed by exposure to 1×10^{-6} Torr ozone at 300 °C for 20 minutes without first depositing K. Supplementary Figure 4 shows an angle integrated valence band spectra of an as-grown Sr_2IrO_4 sample (black), the same sample after an anneal without K (blue) which shows no change and then after K deposition and the same annealing procedure (red) which significantly alters the spectra and shifts it towards the Fermi level. This demonstrates that K is integral to the process of hole doping.

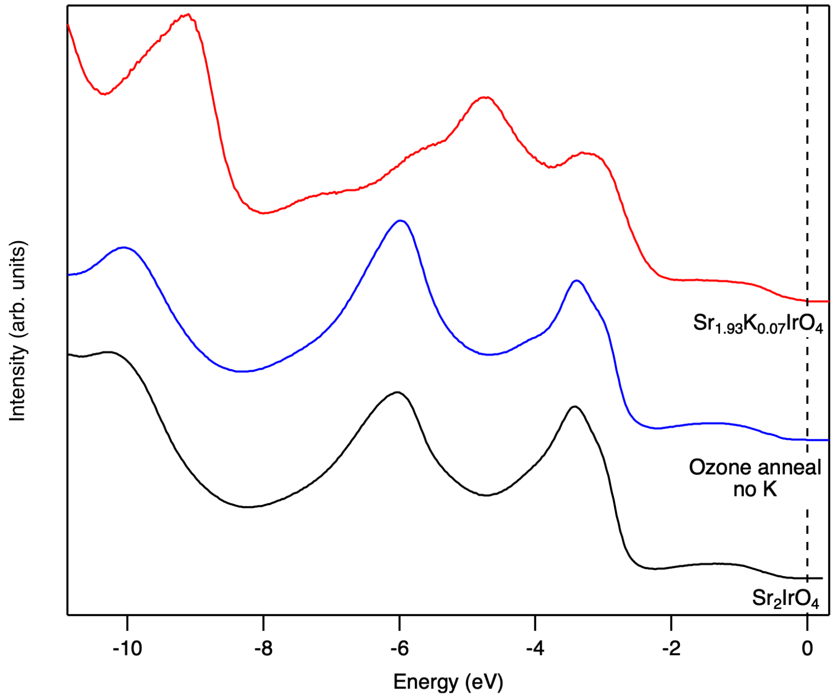

Supplementary Figure 4. Angle-integrated valence band spectra using hν=21.2 eV from an as-grown undoped Sr_2IrO_4 (black), an undoped Sr_2IrO_4 after a 300 °C vacuum then ozone anneal (blue) and a $Sr_{1.93}K_{0.07}IrO_4$ sample that after K deposition was exposed to an identical annealing procedure (red).

Supplementary Note 3: EDC fitting

As discussed in the main text we do not observe a large uniform pseudogap which was reported for $Sr_2Ir_{1-x}Rh_xO_4$ defined by a $\sim$30 meV leading edge midpoint shift[10,11]. In order to quantify this we fit the energy distribution curves (EDCs) of $Sr_{1.93}K_{0.07}IrO_4$ (Supplementary Fig. 5) to a Fermi-Dirac distribution multiplied by a linear term convoluted by a Gaussian function R_ω, with a full width half max of Δ_E, to account for the energy resolution.

$$f(E) = \left[c + \frac{a + b(E - E_\mathrm{F}^\mathrm{S})}{1 + e^{(E - E_\mathrm{F}^\mathrm{S})/k_\mathrm{B}T}} \right] * R_\omega, \tag{1}$$

where a is the height of the Fermi step, b is the slope, c is a constant background, E is energy, E_F^S is the leading edge midpoint shift, k_B is Boltzmann's constant and T is temperature. The Fermi level E_F^Au was independently verified by measurements of a polycrystalline gold reference in electrical contact with the sample. For all momenta we find a difference between the leading edge midpoint E_F^S and the nominal Fermi level E_F^Au of less than 5 meV, which is within

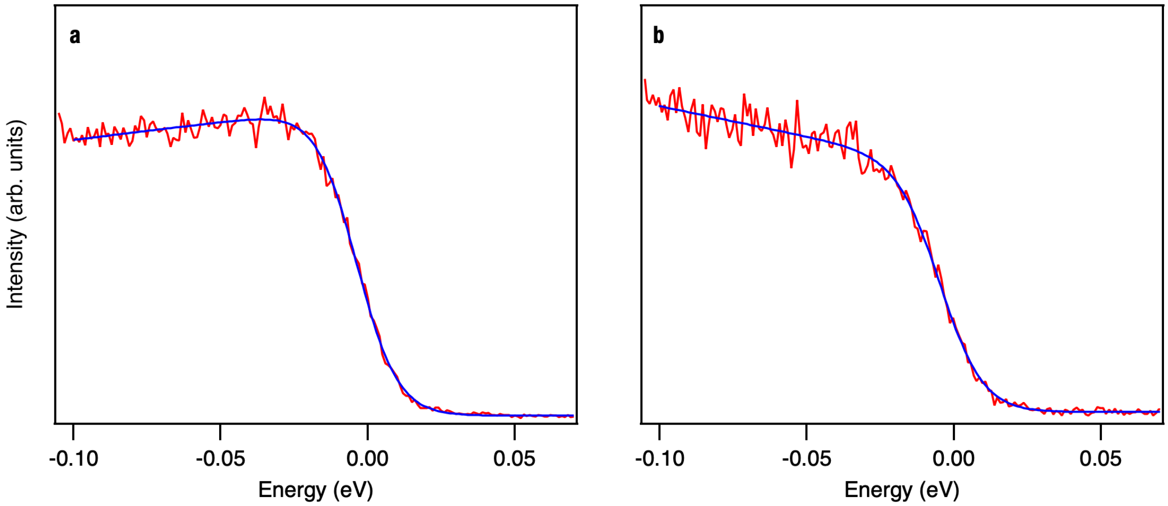

Supplementary Figure 5. Energy distribution curves of $Sr_{1.93}K_{0.07}IrO_4$ reproduced from Fig. 4**e** (red) and fit to Eqn. 1 (blue), fitting parameters are supplied in the main text. **a** EDC at (0, 1.2π). **b** EDC at (π/2,0.6π).

the estimated experimental uncertainty. The fitting parameters used were $\{E_{\mathrm{F}}^{\mathrm{S}}, a, b, T, c, \Delta_E\}$ = $\{-0.0040 \pm 0.0002\ \mathrm{eV}, 0.526 \pm 0.005, 0.60 \pm 0.07, 72 \pm 2\ \mathrm{K}, 0.009 \pm 0.002, 11\ \mathrm{meV}\}$ at (0, 1.2π) in Supplementary Fig. 5a and $\{-0.0043 \pm 0.0004\ \mathrm{eV}, 0.301 \pm 0.006, -0.73 \pm 0.08, 77 \pm 4\ \mathrm{K}, 0.011 \pm 0.002, 11\ \mathrm{meV}\}$ at (π/2, 0.6π) in Supplementary Fig. 5b.

Supplementary Note 4: Tight Binding calculation

The tight binding model, described in the main text, has previously been applied to describe both undoped and electron-doped Sr_2IrO_4 and is described in detail in refs[12–14]. We reproduce the relevant details here for convenience of the reader. The Hamiltonian, $H = H_0 + H_{\mathrm{SO}} + H_U$ includes

$$H_0 = \begin{pmatrix} \Delta_t + e_1(t_1/t_0)^2 & 0 & 0 & -4\gamma l t_1 & 0 & 0 \\ 0 & 0 & 0 & 0 & -2\gamma l t_2 & 0 \\ 0 & 0 & 0 & 0 & 0 & -2\gamma l t_3 \\ -4\gamma l^\dagger t_1 & 0 & 0 & \Delta_t + e_1(t_1/t_0)^2 & 0 & 0 \\ 0 & -2\gamma l^\dagger t_2 & 0 & 0 & 0 & 0 \\ 0 & 0 & -2\gamma l^\dagger t_3 & 0 & 0 & 0 \end{pmatrix}, \quad (2)$$

where $t_0 = 0.35$ eV, $\Delta t = 0.15$ eV is the tetragonal crystal field splitting, $\lambda = 0.57$ eV is the SOC parameter, $e_1 = -1.5$ eV, $t_1 = t_0 cos(k_x/2)cos(k_y/2), t_2 = t_0 cos[(k_x + k_y)/2], t_3 = t_0 cos[(k_x - k_y)/2]$ are hopping parameters for the d_{xy}, d_{yz} and d_{zx} orbitals and $l = e^{-i(k_x+k_y)/2}$ describes the reconstruction due to in-plane octahedral rotation, which as shown in Supplementary Fig. 1 also exist in the $Sr_{1.93}K_{0.07}IrO_4$ sample. The basis used is: $\left\{ \langle d_{A_{xy}\uparrow} \rangle , \langle d_{A_{yz}\downarrow} \rangle , \langle d_{A_{zx}\downarrow} \rangle , \langle d_{B_{xy}\uparrow} \rangle , \langle d_{B_{yz}\downarrow} \rangle , \langle d_{B_{zx}\downarrow} \rangle \right\}$, which are degenerate to the time-reversed pairs: $\left\{ \langle d_{A_{xy}\downarrow} \rangle , \langle d_{A_{yz}\uparrow} \rangle , \langle d_{A_{zx}\uparrow} \rangle , \langle d_{B_{xy}\downarrow} \rangle , \langle d_{B_{yz}\uparrow} \rangle , \langle d_{B_{zx}\uparrow} \rangle \right\}$. γ is a tuning parameter which account for differences between the theory and experiment. We use the same values as ref[14]: $\gamma = 1$ for U= 0 eV and $\gamma = 0.65$ for U = 2 eV. The spin orbit coupling is described by:

$$H_{\text{SO}} = \begin{pmatrix} 0 & \lambda/2 & -i\lambda/2 & 0 & 0 & 0 \\ \lambda/2 & 0 & -i\lambda/2 & 0 & 0 & 0 \\ i\lambda/2 & i\lambda/2 & 0 & 0 & 0 & 0 \\ 0 & 0 & 0 & 0 & \lambda/2 & -i\lambda/2 \\ 0 & 0 & 0 & \lambda/2 & 0 & -i\lambda/2 \\ 0 & 0 & 0 & i\lambda/2 & i\lambda/2 & 0 \end{pmatrix}, \tag{3}$$

and Coulomb repulsion is described by:

$$H_U = \begin{pmatrix} -U \langle n_{A_{xy}\uparrow} \rangle & 0 & 0 & 0 & 0 & 0 \\ 0 & -U \langle n_{A_{yz}\downarrow} \rangle & 0 & 0 & 0 & 0 \\ 0 & 0 & -U \langle n_{A_{zx}\downarrow} \rangle & 0 & 0 & 0 \\ 0 & 0 & 0 & -U \langle n_{B_{xy}\uparrow} \rangle & 0 & 0 \\ 0 & 0 & 0 & 0 & -U \langle n_{B_{yz}\downarrow} \rangle & 0 \\ 0 & 0 & 0 & 0 & 0 & -U \langle n_{B_{zx}\downarrow} \rangle \end{pmatrix}. \tag{4}$$

We solve for $\langle n_{\alpha\sigma} \rangle$ self consistently by numerically diagonalizing the Hamiltonian, calculating the density of states and Fermi level to determine the average occupation for each orbital[15], the on-site energy depends on the occupation of the opposite spin $\langle n_{\alpha\bar{\sigma}} \rangle$, which is equal to $\langle n_{\alpha\sigma} \rangle$ because all bands are doubly degenerate Kramers pairs. For the calculation of undoped Sr_2IrO_4 shown in Fig. 2a and Fig. 4(a-c) $U = 2eV, n_{tot} = 5$, $\left\{ \langle n_{A_{xy}\uparrow} \rangle , \langle n_{A_{yz}\downarrow} \rangle , \langle n_{A_{zx}\downarrow} \rangle , \langle n_{B_{xy}\uparrow} \rangle , \langle n_{B_{yz}\downarrow} \rangle , \langle n_{B_{zx}\downarrow} \rangle \right\} = \{0.882, 0.614, 0.614, 0.981, 0.954, 0.954\}$.

Supplementary References

* kmshen@cornell.edu

[1] Shai, D. E. *et al.* Quasiparticle mass enhancement and temperature dependence of the electronic structure of ferromagnetic $SrRuO_3$ thin films. *Phys. Rev. Lett.* **110**, 087004 (2013).

[2] Uchida, M. *et al.* Correlated vs. conventional insulating behavior in the $j_{eff} = \frac{1}{2}$ vs. $\frac{3}{2}$ bands in the layered iridate Ba_2IrO_4. *Phys. Rev. B* **90**, 075142 (2014).

[3] Nie, Y. F. *et al.* Interplay of spin-orbit interactions, dimensionality, and octahedral rotations in semimetallic $SrIrO_3$. *Phys. Rev. Lett.* **114**, 016401 (2015).

[4] Burganov, B. *et al.* Strain control of fermiology and many-body interactions in two-dimensional ruthenates. *Phys. Rev. Lett.* **116**, 197003 (2016).

[5] Kawasaki, J. K., Uchida, M., Paik, H., Schlom, D. G. & Shen, K. M. Evolution of electronic correlations across the rutile, perovskite, and ruddelsden-popper iridates with octahedral connectivity. *Phys. Rev. B* **94**, 121104 (2016).

[6] Nair, H. P. *et al.* Demystifying the growth of superconducting Sr_2RuO_4 thin films. *APL Materials* **6**, 101108 (2018).

[7] Nair, H. P. *et al.* Synthesis science of $SrRuO_3$ and $CaRuO_3$ epitaxial films with high residual resistivity ratios. *APL Materials* **6**, 046101 (2018).

[8] Liu, Y., Nair, H. P., Ruf, J. P., Schlom, D. G. & Shen, K. M. Revealing the hidden heavy fermi liquid in $CaRuO_3$. *Phys. Rev. B* **98**, 041110 (2018).

[9] Seah, M. P. & Dench, W. A. Quantitative electron spectroscopy of surfaces: a standard data base for electron inelastic mean free paths in solids. *Surf. Interface Anal* **1**, 2–11 (1979).

[10] Louat, A. *et al.* Formation of an incoherent metallic state in Rh-doped Sr_2IrO_4. *Phys. Rev. B* **97**, 161109 (2018).

[11] Louat, A. *et al.* ARPES study of orbital character, symmetry breaking, and pseudogaps in doped and pure Sr_2IrO_4. *Phys. Rev. B* **100**, 205135 (2019).

[12] Jin, H., Jeong, H., Ozaki, T. & Yu, J. Anisotropic exchange interactions of spin-orbit-integrated states in Sr_2IrO_4. *Phys. Rev. B* **80**, 075112 (2009).

[13] Carter, J.-M., Shankar V., V. & Kee, H.-Y. Theory of metal-insulator transition in the family of perovskite iridium oxides. *Phys. Rev. B* **88**, 035111 (2013).

[14] De La Torre, A. *et al.* Collapse of the Mott gap and emergence of a nodal liquid in lightly doped

Sr_2IrO_4. *Phys. Rev. Lett.* **115**, 176402 (2015).

[15] Claveau, Y., Arnaud, B. & Matteo, S. D. Mean-field solution of the Hubbard model: the magnetic phase diagram. *Eur. J. Phys.* **35**, 035023 (2014).
